# Electroacupuncture Regulates Disorders of Gut-Brain Interaction by Decreasing Corticotropin-Releasing Factor in a Rat Model of IBS

**DOI:** 10.1155/2019/1759842

**Published:** 2019-10-13

**Authors:** Ying Chen, Yan Zhao, Dan-ni Luo, Hui Zheng, Ying Li, Si-yuan Zhou

**Affiliations:** Acupuncture and Tuina School, Chengdu University of Traditional Chinese Medicine, Chengdu, Sichuan, China

## Abstract

**Objective:**

Acupuncture is effective for irritable bowel syndrome (IBS); however, the mechanisms of action are not fully understood. We aim to explore the mechanism of electroacupuncture (EA) in the dual regulation of disorders of gut-brain interaction.

**Methods:**

A rat model of IBS was generated by chronic unpredictable mild stress (CUMS). Eight of 32 rats were assigned to the blank control group. The remaining 24 rats received CUMS for 14 days. Then, the rats surviving and successfully modelled were randomly divided into the CUMS group, the CUMS+EA group, and the CUMS+PB (pinaverium bromide) group. In the next 14 days of treatment, rats in the CUMS+EA group were acupunctured at ST25 (*Tianshu*), ST36 (*Zusanli*), SP6 (*Sanyinjiao*), and LR3 (*Taichong*) for 15 min every day. Rats in the CUMS+PB group were treated by the administration of gavage with 2.7 mg/mL pinaverium every day. Visceral pain threshold, the percentage of time spent in open arms (OT%) in the elevated plus maze test (EPMT), and the sucrose preference (SP%) in the sucrose preference test (SPT) were measured at baseline, day 15, and day 30. The expression of zonula occludens-1 (ZO-1), the morphology of the connective structure of intestinal epithelium, the CRF and CRF-R1 mRNA expression in the hypothalamus, and the double staining of intestinal mucosal mast cells (IMMC) and CRF-R1 were measured at the end of the experiment.

**Results:**

Compared with the blank control group, visceral pain threshold pressure, the expression of ZO-1, OT%, SP%, CRF, and CRF-R1 mRNA expression in the hypothalamus, and double staining of IMMC and CRF-R1 were decreased significantly in the CUMS group. Meanwhile, the morphology of the connective structure in the CUMS group was indistinct. Compared with the CUMS group, SP% was significantly increased in the CUMS+EA group, but there was no significant difference for it in the CUMS+PB group. The morphology of the connective structure in the two treatment groups was clear and seeable. And the expression of other parameters mentioned above was apparently increased in the two treatment groups. Compared with the CUMS+PB group, the expression of ZO-1 in the CUMS+EA group was significantly enhanced. And no obvious difference for other parameters was found between the two treatment groups.

**Conclusions:**

EA treatment can decrease the expression of hypothalamic CRF and CRF-R1, relieve anxiety and depression, meanwhile reduce the expression of CRF-R1 in the gastrointestinal mucosa, increase ZO-1 expression, and adjust tight junctions (TJs) to repair the intestinal mucosal barrier. The above roles suggest that EA may play a dual role in alleviating the gastrointestinal and psychological symptoms of IBS, suggesting a potentially dual therapeutic role for EA in regulating disorders of gut-brain interaction in IBS rats.

## 1. Introduction

IBS is a common, chronic gastrointestinal disorder characterized by recurrent lower abdominal discomfort and altered bowel habits [1]. It has become a public health issue because of its high prevalence [2, 3], unclear pathology, and unsatisfactory treatment [4]. Visceral hypersensitivity is the main performance of IBS, which is associated with anxiety, depression, etc. [5]. Furthermore, the latest release of *Rome IV* suggests that the pathogenesis of IBS is mainly due to disorders of gut-brain interaction [6].

A considerable number of studies found that acupuncture can improve gastrointestinal motility, decrease intestinal permeability, and reduce visceral hypersensitivity [7, 8]. Our previous polycentric randomized controlled trial showed that EA is effective against IBS and is superior to loperamide in relieving anxiety [9]. However, the underlying mechanisms of acupuncture regulating both gastrointestinal and psychological symptoms remain to be further studied.

Accumulating evidence has shown that CRF, which is mainly produced in the hypothalamic paraventricular nucleus, can induce altered bowel habits, visceral hypersensitivity, and emotional disorders, such as anxiety-like or depression-like behaviours, by combining with central CRF-R1 [10].

Mast cells (MCs) are important immune cells that are widely distributed in intestinal mucosa. The number and activity of IMMC in IBS patients were both positively correlated with intestinal permeability [11] and abdominal pain [12]. CRF-R1 expression on the cell membrane of IMMC can be activated by oversecreted CRF [13]. Studies have also shown that hyperexpression of CRF leads to TJ changes and increases intestinal permeability [14]. Damage to TJs, the most important protein in intercellular junctions that forms the epithelial barrier, can increase intestinal permeability [15]. ZOs, one of the components of TJs [16], has been found that its decreased expression leads to increased colonic mucosal permeability [17]. Accordingly, the activation of CRF-R1 by oversecreted CRF may increase IMMC expression and intestinal permeability and decrease ZO-1 expression, thus leading to gastrointestinal and psychological symptoms of IBS.

We hypothesized that EA may decrease the expression of CRF and CRF-R1 in the hypothalamus and gastrointestinal mucosa while upregulating ZO-1 expression, adjusting TJs to repair the intestinal mucosal barrier. Thus, EA may play a dual role in alleviating the gastrointestinal and psychological symptoms of IBS and regulating disorders of gut-brain interaction in IBS rats.

## 2. Materials and Methods

### 2.1. Animals and Study Design

SD rats (sex in half, aged 8 weeks, and weighing 250 ± 10 g) were obtained from the Sichuan Academy of Medical Sciences, Chengdu, China. They were housed in individual cages (separation of male and female), maintained on a 12 h light/dark cycle (lights on at 8:00) with food and water available ad libitum. The experiment was approved by the Experimental Animal Welfare Ethics Committee at the Chengdu University of Traditional Chinese Medicine (reference no. 2016-02). The experiments were performed according to the National Guideline for the Care and Use of Laboratory Animals, Amendment 2 (State Council of China, 2013). All efforts were made to minimize suffering.

After 1 week of adaptation, 8 of 32 rats were assigned to the blank control group. The remaining 24 rats received CUMS for 14 days. Colorectal distension (CRD) pressure, OT%, and SP% were taken as markers for model establishment. Then, the rats surviving and successfully modelled were randomly divided into a CUMS group, a CUMS+EA group, and a CUMS+PB group. After 14 days of treatment, the above behavioural tests were performed. All animals were then euthanized under isoflurane anaesthesia. Throughout the entire experiment, animals were housed in groups of 2-4 except when they were subjected to isolation, high-density housing, or behavioural testing.

### 2.2. CUMS Procedure

The CUMS procedure included the following seven stress methods: high-density housing for 24 h, separation of housing for 24 h, restraint for 2 h, 45°C warm swim for 5-10 min, tail pinch for 20 min, inescapable shock for 15 min (2 mA, 30 s on, 270 s off), and 240 Hz shaking-crowding for 1 h [18]. All the methods were applied randomly, and the same method was not continuously applied. During the treatment period, all rats were continuously exposed to stressors except the blank control group.

### 2.3. EA Treatment

Four acupuncture points commonly used in the treatment of IBS were selected. They are ST25, ST36, SP6, and LR3. ST25 is 5 mm lateral to the anterior midline at the navel level. ST36 is located at the posterior and lateral side of the knee joint, 5 mm below the capitulum fibulae. SP6 is located 10 mm above the prominence of the lateral malleolus of the hind limb. LR3 is located between the first and second metatarsal bones on the dorsum of the foot. EA was performed by a well-trained acupuncturist once a day for 14 consecutive days. During treatment, rats were restrained but conscious. Stainless steel disposable acupuncture needles (0.16 × 7 mm, Zhong Yan Tai He Medical Instrument Co., Ltd., Beijing) were inserted at ST25 and ST37 to a depth of 4-5 mm and at SP6 and LR3 to a depth of 2-3 mm and connected to an EA apparatus (HANS-200A, Nanjing, China). Electrical stimulation was applied in the form of disperse-dense waves at alternating 2 Hz and 15 Hz frequencies, respectively, and 1 mA intensity for 15 min [8, 19, 20]. Other animals received only 15 min of restraint stress.

### 2.4. Pinaverium Treatment

Pinaverium (Abbott Healthcare SAS, France) was dissolved in sterile distilled water at a concentration of 2.7 mg/mL and administered by gavage into rats of the pinaverium group at 10 mL/kg once daily. Other animals received only distilled water intragastric administration [21, 22].

### 2.5. Behavioural Assessments of Visceral Hyperalgesia

Rats were deprived of food but had free access to water 24 h before behavioural assessments. The prepared colorectal distension balloon covered with saxoline was completely inserted into the descending colon and rectum under ether anaesthesia. Rats were placed into a transparent acrylic box (20 cm × 8 cm × 6 cm) that could not escape or turn around for 30 min until fully recovered from the anaesthesia. The tube of the balloon was connected via a three-limb tube to a desk model sphygmomanometer. The balloon that inserted into the rat intestinal tract was inflated with air in increments of 5 mmHg at intervals of 5 s until a visible contraction of the abdominal wall was observed. The distension duration and intervals between two distensions were selected for 20 s and 5 min. Five abdominal withdrawal reflex (AWR) scores (AWR 0 to AWR 4) were used to assess the intensity of visceral stimuli [23]: AWR 0: no remarkable behaviour changes; AWR 1: immobility of the rat body or occasionally swing of the head; AWR2: mild abdominal muscle contraction; AWR 3: lifting the abdomen or flatting of the abdomen; and AWR 4: body arching or lifting pelvic structures. The pain threshold was defined as the minimal pressure inside the balloon when the rat showed flatting of the abdomen (AWR 3) during colorectal distension (CRD) [18]. All the measurements were manipulated by two blinded observers, repeated three times, and averaged.

### 2.6. Behavioural Assessment of Anxiety-Depression

#### 2.6.1. Sucrose Preference Test (SPT)

After animals habituated to two water bottles for 3 days in home cages, water bottles were removed 6 h before treatment. A free choice between plain water and 2% sucrose solution was provided to each animal for 12 h. The positions of bottles were counterbalanced across the left or right side of the testing cages. Water intake and sucrose intake were measured during the 12 h dark cycle. The preference for sucrose over water was used as a hedonic measure [18].

#### 2.6.2. Elevated Plus Maze Test (EPMT)

The EPMT was conducted on a plus-shaped apparatus consisting of two 50 × 10 × 1.5 cm open arms and two 50 × 10 × 40 cm enclosed arms elevated 50 cm from the floor. The EPM was conducted in a room illuminated by a single red light bulb over the centre of the maze. Each rat was placed on the maze for 5 min. The apparatus was novel to the rats at the time of testing, and each rat was tested only once. After each rat test, the maze was wiped with ANNE Q (pet deodorant) to prevent odours causing disturbance to the following rats. Sessions were video recorded and scored for (1) time spent in the open arms, (2) time spent in the closed arms, (3) time spent in the central area, and (4) the total time of each subject by an observer blind to the animal's group. A rat was considered to have entered or spent time in an arm only when all four paws were in the respective arm. The percentage of time in the open arms to the total time deducted from the central area was analysed [24, 25].

### 2.7. Tissue Sampling

The hypothalamus and distal colon (3 cm proximal to the anus) were rapidly collected after the rats were executed. The intestinal contents were washed with 0.9% normal saline. Then, the cleaned colon was quickly placed in 4% polyoxymethylene and fixed at room temperature for 48 h for subsequent immunohistochemical analysis. The hypothalamus, located between the posterior margin of the optic chiasma and the anterior pituitary, was wrapped in tinfoil and quickly placed in liquid nitrogen. Then, the hypothalamus was removed from the liquid nitrogen and stored at −80°C for subsequent fluorescence quantitative- (fq-) PCR analysis.

### 2.8. Measurement of CRF and CRF-R1 mRNA in the Hypothalamus by PCR

Using TriPure Isolation Reagent (Roche, Germany) according to the instructions to extract total CRF RNA and CRF-R1 RNA from hypothalamus tissue, RNA quantity was assessed using a NanoDrop 2000 ultramicrospectrophotometer (Thermo, USA). cDNA was synthesized using a reverse transcriptase kit (Roche, Germany). Sequences of gene-specific PCR primers (Shanghai Sangon Biotech Co. Ltd., China) used were as follows: CRF mRNA: forward: 5′-TCTCTGGATCTCACCTTCCACCTT-3′; reverse: 5′-AGTTTCCTGTTGCTGTGAGCTTGC-3′; CRF-R1 mRNA: forward: 5′-TCGGGAGAAGGCTACCAGAC-3′; reverse: 5′-GGCTTCGCACCCTTCCG-3′; and *β*-actin mRNA: forward: 5′-ACAACCTTCTTGCAGCTCCTC-3′; reverse: 5′-CTGACCCATACCCACCATCAC-3′. The fq-RT-PCR of CRF mRNA and CRF-R1 mRNA was performed in 20 *μ*L volumes with SYBR Green PCR Master Mix (Roche, Germany) at 95°C for 2 min and 40 cycles at 95°C for 15 s, 60°C and 60°C for 15 min, using a LightCycler 480 (Roche, Germany).

### 2.9. Immunohistochemistry

The colon tissues were postfixed in 4% paraformaldehyde and immersion fixed for 72 h, embedded in paraffin, and then sliced into 5 *μ*m thick sections. After being washed in TBS, sections were incubated for 30 min in 0.5% Triton X-100 and blocked with 10% goat serum in TBS for 2 h at 37°C.

#### 2.9.1. Double Labelling of CRF-R1 and IMMC

The sections were incubated for 1 h at 37°C with Goat anti-CRHR1/CRF-R (aa250-263, Art. No. 07553, Everest Biotech, UK) and Rabbit Anti-Mast Cell Tryptase antibody (Art. No. 196772, Abcam, USA) diluted in TBST with 10% goat serum before incubating overnight at 4°C. The following day, the sections were washed and incubated with donkey anti-goat IgG H&L (Alexa Fluor® 488, Art. No. 150129, Abcam, USA) and donkey anti-rabbit IgG H&L (Alexa Fluor® 647, Art. No. 150075, Abcam, USA), diluted in TBS with 10% goat serum, for 1 h at 37°C. After washing, the sections were coverslipped with Antifade Mounting Medium and examined at 200x magnification under a fluorescence microscope (Olympus BX51, Japan).

#### 2.9.2. The Expression of ZO-1

The sections were incubated for 1 h at 37°C with anti-ZO-1 (Art. No. 96587, Abcam, USA) diluted in TBST with 10% goat serum before incubating overnight at 4°C. The following day, the sections were washed and incubated with biotin-labelled secondary antibody, diluted in TBS with 10% goat serum, for 1 h at 37°C. After washing, the sections were coverslipped with Antifade Mounting Medium and examined at 400x magnifications by an optical microscope (Leica DM1000, Germany).

### 2.10. Transmission Electron Microscopy (TEM)

After colon tissue was cut into 1 × 1 × 1 mm, the sample was prefixed in 2.5% glutaraldehyde at 4°C for 2 h and then rinsed with 0.1 M phosphate buffer 3 times for 45 min each time. The sample was then postfixed with 1% osmium tetroxide for 1 h and was rinsed as described before and was dyed with 1% uranyl acetate for 2 h. After dehydration with acetone, tissues were soaked in a solution of acetone and epoxy resin and then embedded polymerization at 45°C for 3 h and 65°C for 48 h, respectively. The sample was cut into ultrathin sections (70 nm) and stained with uranyl acetate for 40 min and then lead citrate for 15 min. The samples were examined under TEM (Hitachi HT7700, Japan) for ultrastructural analysis of colon tissue.

### 2.11. Statistical Analysis

Statistical analysis was conducted using SPSS V.15.0 Software (SPSS Inc., Chicago, Illinois, USA). The Shapiro-Wilk test was used to test for the normality of data. Normally and nonnormally distributed data are presented as the mean ± SD and median (IQR), respectively. One-way analysis of variance (ANOVA) followed by a post hoc least significant difference (LSD) test was used for normality assumption and homogeneity of variance data. A nonparametric Kruskal-Wallis test was applied when the normality assumption and homogeneity of variance were violated. The Duncan and Mann-Whitney *U* tests were used for pairwise comparisons following parametric and nonparametric tests, respectively. *p* < 0.05 was considered statistically significant.

## 3. Results

### 3.1. Baseline Characteristics

One rat died after 17 days, which resulted in the following final sample sizes: blank control group (*n* = 8), CUMS group (*n* = 7), CUMS+EA group (*n* = 8), and CUMS+PB group (*n* = 8). There were no other fatalities or exclusions from the study.

### 3.2. Effect of EA on Visceral Hyperalgesia

**Figure 1:**
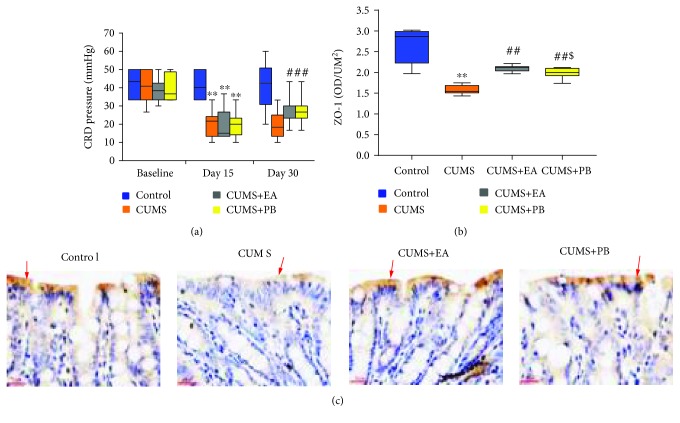
Pain threshold pressure measured by CRD (a), the mean optical density of ZO-1 (b). Data were expressed as the median (IQR). ^∗∗^*p* < 0.01 vs. blank control group, ^##^*p* < 0.01 vs. CUMS group, and ^$^*p* < 0.01 vs. CUMS+EA group. Immunohistochemical staining of ZO-1 (×400) (c).

As shown in Figure 1(a), there was no significant difference in CRD pressure between the four groups at baseline (43.34 (16.67), 40.84 (16.68), 38.35 (9.14), and 36.67 (15.42), *p* > 0.05). After CUMS, CRD pressure was markedly decreased in the other three groups compared with the blank control group (21.67 (10.84) vs. 40.00 (16.67), *p* < 0.01; 15.00 (13.34) vs. 40.00 (16.67), *p* < 0.01; and 20.00 (9.16) vs. 40.00 (16.67), *p* < 0.01). Additionally, CRD pressure was significantly elevated in the CUMS+EA and CUMS+PB groups compared with the CUMS group after treatment (23.33 (6.67) vs. 18.34 (11.67), *p* < 0.05; 26.67 (6.67) vs. 18.34 (11.67), *p* < 0.01). No significant difference was found between the two receiving treatment groups (23.33 (6.67) vs. 26.67 (6.67), *p* > 0.05).

### 3.3. Effects of EA on the Intestinal Epithelial Barrier TJs

#### 3.3.1. The Mean Optical Density of ZO-1

As shown in Figure 1(b), the mean optical density of ZO-1 in the intestinal epithelial barrier was significantly inhibited in the CUMS group compared with the blank control group (1.54 (0.18) vs. 2.87 (0.76), *p* < 0.01). The CUMS+EA and CUMS+PB groups both significantly improved the mean optical density of ZO-1 compared with the CUMS group (2.12 (0.10) vs. 1.54 (0.18), *p* < 0.01; 2.00 (0.18) vs. 1.54 (0.18), *p*<0.01). Furthermore, the mean optical density of ZO-1 in the CUMS+EA group increased more obviously than the CUMS+PB group (2.12 (0.10) vs. 2.00 (0.18), *p*<0.01).

#### 3.3.2. Immunohistochemical Staining of ZO-1

As shown in Figure 1(c), there were much sepia ZO-1 positive expression on the intestinal epithelial top in the blank control group. However, the positive expressions of ZO-1 in the CUMS group were significantly less than in the blank control group, and the colour of the CUMS group was markedly lighter. The number and colouring depth of ZO-1 positive expressions in the CUMS+EA and CUMS+PB groups were between the blank control group and the CUMS group.

#### 3.3.3. Effect of EA on the Morphology of the Connective Structure of the Intestinal Epithelium under TEM

**Figure 2:**
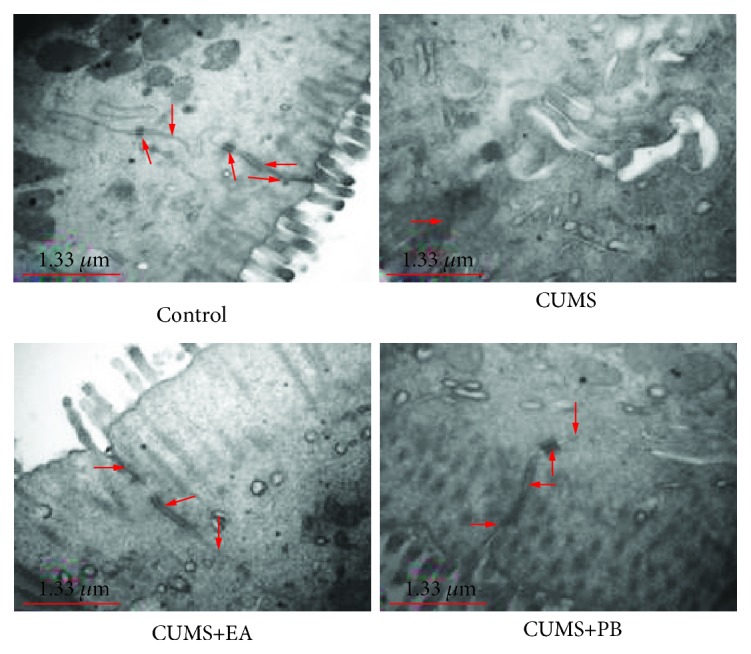
Morphology of the connective structure of the intestinal epithelium under TEM (×30000). Scale bar = 1.33 *μ*m.

As demonstrated in Figure 2, the TJs, adherence junctions, gap junctions, and desmosomes in the intestinal epithelial cells of the blank control group were clear and distinct under TEM. While in the CUMS group, the structure of TJs was indistinct with no adherence junctions, gap junctions, and desmosomes. In the CUMS+EA group, TJs and adherence junctions had clear and seeable structures, and the number of desmosomes was less than that of the blank control group. In the CUMS+PB group, the adherence junctions, gap junctions, and desmosomes were clear under TEM; yet, the gap of TJs was wider than that of the blank control group.

### 3.4. Effect of EA on Anxiety and Depression-Like Behaviours

**Figure 3:**
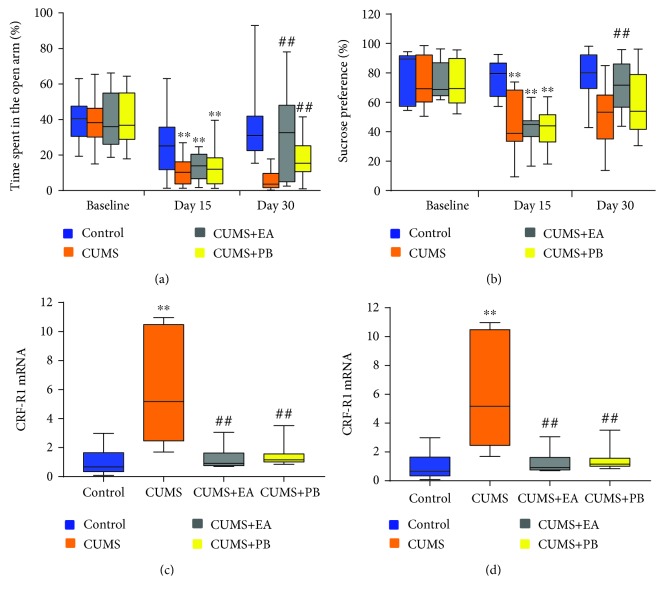
The percentage of time spent in the open arms in the EPMT (a) and SP% (b) in the baseline, 15th day, and 30th day; the expression of CRF in the hypothalamus (c) and the expression of CRF-R1 mRNA in the hypothalamus (d). Data in (a), (b), (c), and (d) were expressed as the median (IQR). ^∗∗^*p* < 0.01 vs. blank control group, ^#^*p* < 0.05 vs. CUMS group, and ^##^*p* < 0.01 vs. CUMS group.

As shown in Figure 3(a), no significant difference was found in OT% between the four groups during baseline (40.37 (17.03), 38.21 (16.21), 35.99 (28.74), and 36.76 (26.11), *p* > 0.05). All groups receiving CUMS displayed a statistically significant decrease in OT% compared to the blank control group (10.37 (12.48) vs. 29.10 (21.70), *p* < 0.01; 13.94 (13.86) vs. 29.10 (21.70), *p* < 0.01; 11.99 (14.54) vs. 29.10 (21.70), *p* < 0.01). After treatment, the OT% was markedly elevated in the CUMS+EA and CUMS+PB groups compared with the CUMS group (32.60 (43.12) vs. 3.67 (8.05), *p* < 0.01; 15.43 (14.62) vs. 3.67 (8.05), *p* < 0.01). There was no significant difference between the two treatment groups (32.60 (43.12) vs. 15.43 (14.62), *p* > 0.05).

As shown in Figure 3(b), no significant difference was found in SP% between the four groups during baseline (89.44 (34.26), 69.27 (31.88), 68.78 (22.31), and 69.39 (30.32), *p* > 0.05). After modelling, the SP% in the other three groups had a significant reduction compared with the blank control group (38.91 (34.77) vs. 79.51 (22.57), *p* < 0.01; 44.93 (10.72) vs. 79.51 (22.57), *p* < 0.01; and 44.06 (18.58) vs. 79.51 (22.57), *p* < 0.01). After the treatment, the SP% of the CUMS+EA and CUMS+PB groups were obviously higher than the CUMS group (71.69 (29.39) vs. 53.35 (29.77), *p* < 0.01; 53.92 (37.35) vs. 53.35 (29.77), *p* > 0.05). No significant difference was found between the two receiving treatment groups (71.69 (29.39) vs. 53.92 (37.35), *p* > 0.05).

### 3.5. Effects of EA on CRF and CRF-R1 in the Hypothalamus

As shown in Figure 3(c), the relative expression of CRF in the hypothalamus was significantly elevated in the CUMS group compared with the blank control group (1.75 (0.77) vs. 0.92 (0.40), *p* < 0.01). CUMS+EA and CUMS+PB both obviously inhibited CRF expression compared with the CUMS group (1.25 (0.45) vs. 1.75 (0.77), *p* < 0.01; 1.02 (0.58) vs. 1.75 (0.77), *p* < 0.01). No significant difference was found between the CUMS+EA and CUMS+PB groups (1.25 (0.45) vs. 1.02 (0.58), *p* > 0.05).

As shown in Figure 3(d), the expression of CRF-R1 in the hypothalamus was markedly improved in the CUMS group compared with the blank control group (5.18 (8.02) vs. 0.67 (1.29), *p* < 0.01). CUMS+EA and CUMS+PB both significantly suppressed CRF-R1 expression compared with the CUMS group (0.92 (0.87) vs. 5.18 (8.02), *p* < 0.01; 1.15 (0.55) vs. 5.18 (8.02), *p* < 0.01). There was no significant difference between the CUMS+EA and CUMS+PB groups (0.92 (0.87) vs. 1.15 (0.55), *p* > 0.05).

### 3.6. Effects of EA on CRF-R1 and MC in Gastrointestinal Mucosa

**Figure 4:**
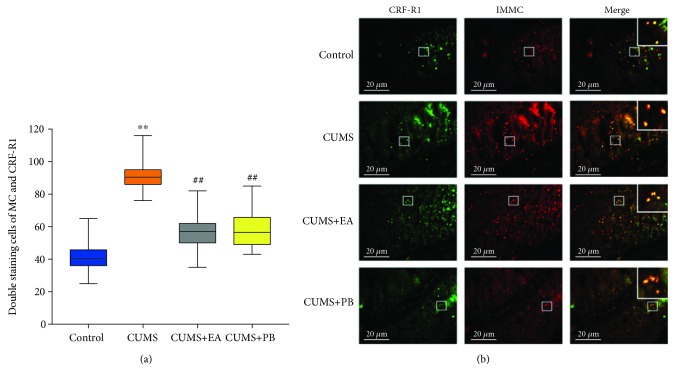
Double staining of MC and CRF-R1 in gastrointestinal mucosa (a). Data were expressed as median (IQR). ^∗∗^*p* < 0.01 vs. blank control group, ^##^*p* < 0.01 vs. CUMS group. Double staining cells of MC and CRF-R1 in gastrointestinal mucosa (×200) (b). Scale bar = 20 *μ*m.

As shown in Figure 4(a), the double immunostaining of cells for MC and CRF-R1 in gastrointestinal mucosa was markedly increased in the CUMS group compared with the blank control group (90.50 (9.00) vs. 40.50 (9.75), *p* < 0.01). CUMS+EA and CUMS+PB both significantly reduced the number of cells double stained for CRF-R1 and MC compared with the CUMS group (57.00 (12.00) vs. 90.50 (9.00), *p* < 0.01; 56.50 (16.75) vs. 90.50 (9.00), *p* < 0.01). No significant difference was found between the CUMS+EA and CUMS+PB groups (57.00 (12.00) vs. 56.50 (16.75), *p>*0.05).

To evaluate the association between MC and CRF-R1 in gastrointestinal mucosa, double labelling of MC and CRF-R1 was performed on sections of the rat gastrointestinal mucosa. A high proportion of CRF-R1 colocalized with MC was found in colon mucosa. CRF-R1 immunoreactivity within cell bodies showed green cytoplasmic staining, and positive IMMC staining was observed in red, while yellow was the double staining of CRF-R1 and IMMC, as demonstrated in Figure 4(b).

## 4. Discussion

It is pointed out in *Rome IV Standards of Functional Gastrointestinal Disorders* that IBS is the disorder of gut-brain interaction, further elaborating the importance of the interinfluence between intestinal and emotional symptoms in IBS [26]. Visceral hypersensitivity is the main performance of IBS and one of its pathophysiologic causes. Additionally, 20% to 60% of IBS patients have mental disorders such as anxiety and depression, which are positively correlated with IBS [27].

AWR is a major indicator of intestinal sensitivity. A previous study found that the pain threshold of IBS patients was obviously lower than that of healthy patients when the duodenum received intense distention stimulation [28]. In this study, we used the corresponding sphygmomanometer reading of the AWR pain threshold (AWR 3) as the indicator of visceral sensitivity. The results also showed that the visceral pain threshold of IBS rats decreased substantially after CUMS. Additionally, EA and PB treatments effectively reduced visceral hypersensitivity without obvious differences. This finding is consistent with other studies showing that acupuncture can reduce visceral hypersensitivity, decrease intestinal permeability, and improve gastrointestinal motility [7, 8]. In addition, the effect of EA is equal to PB, the guide-recommended medicine.

Damage to TJs could destroy the intestinal epithelial barrier and increase intestinal permeability [29, 30]. ZO-1 expression in the IBS patients' colon is distinctly reduced and inversely proportional to intestinal mucosa permeability [31]. This research found that ZO-1 expression in the colon was decreasing and the TJ structure under TEM was indistinct with no adherence junctions, gap junctions, and desmosomes, showing impaired intestinal epithelial barrier and increased mucosal permeability, which is consistent with the aforementioned reports. In our study, EA and PB effectively improved colonic ZO-1 expression and recovered the intestinal epithelial barrier, and EA was superior to PB, which was similar to HX Shang's: after 12 weeks of acupuncture treatments on Crohn's disease, ZO-1 expression in the intestinal epithelial was increased compared to mesalazine [32]. Furthermore, the number and colouring depth of ZO-1 immunohistochemical staining are better by the treatment of EA and PB. Improved TJs and adherence junctions by EA were seen clear under TEM, with improved desmosome not being clear; meanwhile, adherence junctions, gap junctions, and desmosomes with PB had visible structures; yet, the improvement of the TJs was not clearly visible. A previous experiment found that haemorrhaged rats treated with EA showed a robust structure of ZO-1 and had significantly higher ZO-1 expression than the model groups [33]. Our result is similar to theirs. Consequently, EA improves the intestinal epithelial barrier and reduces the permeability of intestinal mucosa by enhancing the expression of ZO-1 in TJs.

As for mental disorders, rats' anxiety-like behaviours were evaluated by OT% of EMPT and depression-like behaviours by SP%. In this experiment, OT% and SP% of rats decreased significantly after modelling, and both EA and PB treatments can effectively increase OT%. EA can distinctly improve SP% to a relatively normal level, while PB had no effective influence on it. As a result, EA may be superior to PB in improving depression-like behaviours, although there was no significant difference between the two treatments. This result is consistent with the following finding: EA can enhance the SP% of depressing rats and relieve rats' anxiety and depression-like behaviours, with a similar effect to PB, an antidepressant medicine [34].

According to previous studies, central CRF-R1 activation is related to growing mental disorders, visceral hypersensitivity, and intestinal movements [35]; intestinal CRF-R1 activation is relevant to abdominal pain caused by intestinal movements and growing visceral hypersensitivity [13, 36]. Moreover, activated intestinal CRF-R1 can increase the content of IMMC. Our study observed that the expression levels of CRF and CRF-R1 mRNA in the hypothalamus, CRF-R1 in the gastrointestinal mucosa, and IMMC in IBS rats were apparently increasing. Both EA and PB can effectively decrease CRF and CRF-R1 expression in the hypothalamus and downregulate CRF-R1 and IMMC expression in the gastrointestinal mucosa. EA was better than PB in weakening CRF and CRF-R1 expression in the hypothalamus, while they were equally effective in reducing the expression of CRF-R1 and IMMC. As a result, EA was effective in downregulating CRF-R1 expression in the hypothalamus and IMMC, decreasing the expression of hypothalamus CRF, alleviating rats' visceral hypersensitivity and anxiety and depression-like behaviours, and meeting with Wu HG's research [37]. He found that EA could weaken IBS rats' visceral hypersensitivity, which was related to MC decrease and degranulation ratio of colon mucosa.

In conclusion, our study indicates that EA can decrease the expression of hypothalamic CRF and CRF-R1, relieve mental disorders, meanwhile reduce the expression of CRF-R1 in the gastrointestinal mucosa, decrease IMMC, increase ZO-1 expression, and adjust TJs to repair the intestinal mucosal barrier, suggesting a potentially dual therapeutic role for EA in alleviating the gastrointestinal and psychological symptoms of IBS, meaning that EA may regulate disorders of gut-brain interaction in IBS rats.

## Data Availability

The data used to support the findings of this study are available from the corresponding author upon request.
